# La neurofibromatose de type I

**DOI:** 10.11604/pamj.2014.17.50.2822

**Published:** 2014-01-23

**Authors:** Kbira El Morabite, Baderddine Hassam

**Affiliations:** 1 Service de Dermatologie, CHU Ibn Sina, Université Med V, Souissi, Rabat, Maroc

**Keywords:** Neurofibromatose de type I, maladie de Recklinghausen, génodermatose, Neurofibromatosis type I, Recklinghausen disease, genodermatosis

## Image en medicine

La neurofibromatose de type 1 (NF1) ou maladie de Recklinghausen est une génodermatose autosomique dominante, elle touche de 1/3000 à 1/4000 personnes. La NF1 est caractérisée par une variabilité de son expression clinique qu'on peut retrouver au sein de la même famille. Des critères de diagnostic ont été établis en 1988. Le diagnostic de la NF1 est porté quand au moins deux des signes suivants sont rencontrés : Six taches Café au lait, des lentigines axillaires ou inguinales, deux neurofibromes cutanés ou un neurofibrome plexiforme, deux nodules de Lisch (harmartomes iriens), une lésion radiologique spécifique (dysplasie du sphénoïde, une pseudarthrose, dysplasie de la corticale des os longs), un gliome des voies optiques et un parent du premier degré atteint. Le risque tumoral et l'évolution imprévisible de la maladie imposent une surveillance régulière qui repose essentiellement sur l'examen clinique. Nous rapportons le cas d'une patiente âgée de 54 ans sans antécédents pathologiques particuliers, chez qui le diagnostic de la NF1 était retenu sur la présence d'une vingtaines tâches café au lait, des lentigines disséminées au corps, des neurofibromes cutanés et sous cutanés de taille variable et des nodules de Lish nombreux à l'examen ophtalmologique. La patiente a bénéficié d'un bilan radiologique fait de radiographie osseuse et une TDM cérébrale n'ayant pas objectivé d'anomalies. Une surveillance clinique est préconisée chez elle tous les 6 mois à la recherche de complications tumorales et leur prise en charge précoce.

**Figure 1:**
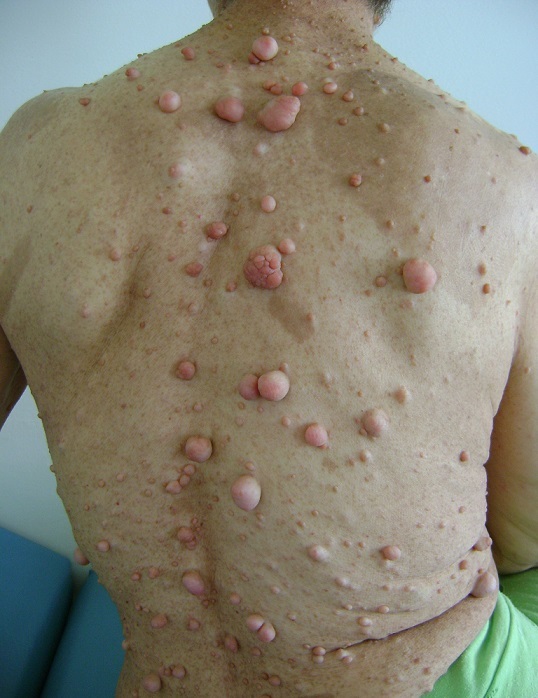
Nombreux neurofibromes cutanés et sous cutanés de taille variable

